# “Accessible and Acceptable” Drop-In Mental Health Services for Children With Long-Term Conditions: A Mixed-Methods Exploratory Analysis of Integrated Services From a Health Equity Perspective

**DOI:** 10.1177/13591045261473984

**Published:** 2026-08-09

**Authors:** Émer Kenny, Anna Roach, Nyah Patel, Sophie Bennett, Isobel Heyman, Anna Coughtrey, Roz Shafran

**Affiliations:** 1 UCL Great Ormond Street Institute of Child Health, London, UK; 2 UCL Division of Psychology and Language Sciences, London, UK; 3 Department of Psychology, 34426 King’s College London, Institute of Psychiatry Psychology and Neuroscience, London, UK; 4 Cambridge Children’s Hospital Project Team and Paediatric Psychological Medicine Service, Addenbrooke’s Hospital, Cambridge, UK

**Keywords:** long-term conditions, drop-in mental health service, integrated care, accessibility, acceptability, physical-mental comorbidity, mixed-methods, health equity, ethnic minority access

## Abstract

**Introduction:**

Children and young people (CYP) with long-term physical health conditions (LTCs) are at increased risk of emotional and behavioural difficulties, yet barriers to psychological support remain, particularly for families from ethnic minority backgrounds. Mental health drop-in services were set up and evaluated at 6 paediatric healthcare settings, aiming to provide a more accessible route to care.

**Methods:**

This study conducted a secondary analysis of quantitative and qualitative data collected as part of the multi-site roll-out of mental health drop-in services in paediatric healthcare settings to explore their accessibility and acceptability. The services provided short-term psychological interventions delivered by child wellbeing practitioners or equivalent, under supervision of a clinical psychologist 120 families provided quantitative data, and 104 families shared qualitative feedback. Differences in access routes and mental health symptoms were compared across White British families and those from different ethnicities. Inductive, latent content analysis of the open text feedback was conducted to understand families’ experiences of the drop-in services.

**Results:**

CYP from ethnic minority backgrounds were over-represented in the present study (32%), when compared with nationwide CAMHs users that identify as non-white British (21%). Families from ethnic minority backgrounds reported comparable mental health outcomes and satisfaction levels to White British families. There was a significant difference in mental health change scores, with ethnic minority families showing greater improvements after accessing the service, t (46) = −3.05, p < .01, d = −1.08. Qualitative analysis found that the services were highly acceptable across ethnicities, highlighting themes of life-changing support, therapeutic relationships, and improved parent and family wellbeing.

**Discussion:**

Drop-in services embedded in paediatric healthcare settings are both accessible and acceptable, demonstrating positive clinical outcomes, and positive family feedback across different ethnic groups. This model may help reduce inequalities by improving access to psychological support for ethnic minority families with CYP living with LTCs.

## Plain Language Summary

Why was the study done? Children and young people with long term illnesses are more likely than others to have poor mental health. However, these children and their families usually struggle to access mental health support. Receiving support can be even harder for families from non-White backgrounds. A drop-in service providing short-term mental health support for these children was set up at six children’s hospitals across England. This study looked at how families from non-White backgrounds accessed these services, and whether it helped their mental health. It also explored what all families, across ethnicities, thought of the service. What did the researchers do? Researchers looked at the mental health of 120 families before and after using the service. They explored if families from non-White backgrounds had the same experience as White British families who used the service. Families gave feedback about the service. This was used to understand what families thought of the service, what they liked about it and what they thought could be improved. What did the researchers find? Researchers found that many families who were not White used the service. Non-White families’ mental health symptoms improved more than White families’ after using the service. Families from all backgrounds shared very positive experiences with the service, mainly focusing on how it changed children's lives, improved parents' mental health, and how it was an easy way to get support. What do the findings mean? Families from all backgrounds shared positive experiences reaching and using the service. Providing mental healthcare this way at children's hospitals may make it easier for children with long term illnesses and families from all ethnicities to receive mental health support.

## Introduction

Approximately 1.7 million (23%) of children and young people (CYP) in England live with a long-term physical health condition (LTC) (Brooks et al., 2015; NICE, 2020). LTCs can be defined as physical health conditions for which a cure is unlikely, that require ongoing management and increased use of health services (Moore et al., 2019). CYP with LTCs are significantly more likely to develop mental health symptoms than their healthy peers, with research suggesting around 50% of CYP with LTCs meet diagnostic criteria for at least one mental health disorder, compared with around 16% of the general population (Butler et al., 2018). CYP with LTCs also report increased loneliness, decreased social functioning and reduced health-related quality of life (Bateman et al., 2023; Hall et al., 2019; McGlone and Long, 2020). These adverse outcomes are also reported by their wider families, with siblings and parents of children with LTCs facing increased stress alongside mental and physical health difficulties (Cohn et al., 2020; McKenzie Smith et al., 2018).

Previous research has indicated that individuals from ethnic minority backgrounds are at higher risk of developing multiple LTCs, that such conditions develop earlier when compared with the majority white population in the UK, and that these families report greater difficulties accessing support (Ekezie et al., 2023; Hayanga et al., 2024). In this paper, the term *ethnic minority*^1^ refers to all ethnic groups except the white British group” (GOV.UK, 2021). Additional research suggests that ethnic minority families may be at an increased risk of developing mental health problems (Bamford et al., 2021) and show poorer treatment outcomes and reduced access to support (Cooper et al., 2013; Ruphrect-Smith et al., 2023).

Poor access to healthcare reported by families from ethnic minority backgrounds is consistent with the wider challenge of accessing evidence-based treatment for CYP with mental health disorders and LTCs despite evidence-based treatments showing effectiveness for this population (Catanzano et al., 2020). LTCs often exacerbate common barriers to accessing care, with families facing heightened healthcare costs and logistical issues due to the burden of navigating physical and mental healthcare systems simultaneously (Byrne et al., 2023; Macdonald et al., 2021; Naylor et al., 2012). Physical health and mental health services often operate separately, with different referral routes, staff and locations (King et al., 2024). These challenges are likely compounded for CYP from ethnic minority backgrounds, who experience additional, significant barriers accessing healthcare services – such as structural inequalities, cultural stigma and discrimination in healthcare (Danso & Danso, 2021; Garney et al., 2021; Nwokoroku et al., 2022). It is reported that CYP from ethnic minority backgrounds are less likely to contact a general practitioner regarding their mental health, or to receive primary mental healthcare support (Kapadia et al., 2022), with families reporting preferences of accessing support in places they regularly frequent, such as community centres or schools (Coelho et al., 2022).

Such barriers may be overcome by providing multiple entry points to support (i.e. clinician or self-referral), in a more-familiar environment.

One way of increasing access to psychological interventions for CYP with LTCs more widely is to integrate mental health support within physical health settings. A drop-in service, named “The Lucy Project”, was initially trialled at a specialist paediatric hospital which provided ‘low intensity’ psychological interventions such as guided self-help, signposting or referral to appropriate services (Catanzano et al., 2021; Shafran et al., 2021). Families could drop-in or self-refer, or could be referred to the services by their clinical team. This service was found to reduce emotional and behavioural symptoms and improve quality of life in young people with LTCs (Catanzano et al., 2021). It was also found to improve parental and sibling mental health (Bennett et al., 2021, 2023), was highly acceptable to families (Catanzano et al., 2023) and was cost effective (Clarke et al., 2022). There was initial evidence that the drop-in model improved accessibility to mental health support for families from ethnic minority backgrounds (Catanzano, Bennett, Kerry, et al., 2021) as families from Asian and Black backgrounds were over-represented in the study sample when compared to nationwide data from child and adolescent mental health services (CAMHS). Taken together, these findings suggest that integrated care via a drop-in service may be effective in increasing access to mental health support for ethnic minority families with LTCs.

This model of drop-in services, offering low intensity mental health support at point of need was subsequently adopted at six paediatric healthcare settings across England (Roach et al., 2024). An evaluation of 120 participants found significant reductions in emotional and behavioural symptoms (p < 0.01; Cohen’s d = 0.39) and improved quality of life (p < 0.01; Cohen’s d = 0.44) across CYP and their parents (Roach et al., 2025).

The aim of the current study was to use the data from the national roll-out study (Roach et al., 2025) to further understand both the accessibility and acceptability of the drop-in model, both of which are critical for implementing new services (Peterson et al., 2023). Accessibility, defined as the opportunity to reach and avail of services during situations of high need (Levesque et al., 2013), is particularly important to explore from the perspective of families from ethnic minority backgrounds, as adverse experiences with referral routes have been linked to poorer treatment outcome experiences and longer durations of untreated disorders (Clement et al., 2015; Hauenstein et al., 2019; Ruphrect-Smith et al., 2023). Therefore, we first sought to understand who accessed the service, specifically considering differences in access routes and mental health symptoms reported by families from different ethnic groups. Second, we explored how families perceived the service, investigating acceptability through understanding families’ qualitative experiences of the drop-in services.

## Methods

### Participants

The present research was part of a larger prospective single-arm, multi-site interventional trial (ISRCTN15063954) where families were recruited across six NHS sites in England: Cambridge and Peterborough Trust, University College London Hospital, Hinchingbrooke Hospital, Sheffield Children’s Hospital, Leeds Teaching Hospital and Peterborough City Hospital. The study protocol, and main evaluation paper provide further information on recruitment, methods and outcome measures (Roach et al., 2024, 2025).

Study participants had to be a patient, aged between 0-25 years, at a participating service for at least 6 months, or be a carer/family member of such patient. CYP and their families could self-refer to the service using the QR codes or email address located on posters at participating sites. Alternatively, CYP and families could also be referred by their clinician or direct care team, who, upon receiving their consent to contact, could share contact details with the study team.

#### Intervention

CYP and their families were offered an initial assessment within two weeks after consenting, to ensure “drop-in” support and timely access to treatment. Following the assessment and discussion with a supervisor, CYP with mild to moderate emotional and/or behavioural difficulties were offered low intensity CBT interventions (for anxiety, low mood or challenging behaviour). If these interventions were not clinically appropriate (e.g. if their concern surrounded adjustment to new condition or a bereavement) individuals were referred to other therapy modalities (such as narrative therapy, family therapy or counselling) or signposted towards other services (e.g. a local support group or charity). Parents accessing help for their own mental health were offered a one-off CBT session or referred or signposted for further support.

## Measures

Demographic data were collected from 120 families (120 CYP and 94 parents) at baseline. Ethnicity, gender and first language spoken were self-reported for CYP aged 12 years and older. Parents reported ethnicity for CYP under 12 years old and self-reported their own ethnicity, gender, first language and number of CYP in their household. UK census categories from 2021 were used (Office for National Statistics, 2022)^2^.

Four mental health outcome measures were collected and are detailed below. Self-reported and parent-reported measures were used depending on age of the CYP.

(1) The Strengths and Difficulties questionnaire (SDQ) is a 25-item survey used to measure common emotional and behavioural symptoms in young people (Goodman, 1997, 1999, Goodman and Goodman, 2011). It assesses five subscales: prosocial, hyperactivity, peer problems, emotional problems and conduct problems, and calculates a ‘total difficulties’ score. Each statement is rated on a 3-point Likert Scale of 0 (not true), 1 (somewhat true) and 2 (certainly true). The total difficulties score of the SDQ ranges from 0 to 40 and total scores of 17 and above are above clinical threshold on the parent-reported measures, and 20 and above for self-reported total scores. The SDQ has shown moderate test-retest reliability and good concurrent, convergent and discriminant validity (Goodman and Goodman, 2011). Self-reported SDQ questionnaires were completed by CYP 11 years and older, and parents completed the parent-reported SDQ based on their CYP’s symptoms.
(2) The Pediatric Quality of Life Inventory (PedsQL) is a 23-item questionnaire which has four scales: physical, emotional, social, and school functioning (Varni et al., 1999, 2001). Each question asks how much a problem something is and is scored on a 5-point Likert scale from: 0 (never) to 4 (almost always). Scores are then transformed on a scale of 0-100 where higher scores indicate better quality of life. Self-reported PedsQL questionnaires were completed by CYP 8 years and older, and parents completed the parent-reported PedsQL based on their CYP’s wellbeing.
(3) The Generalised Anxiety Disorder Questionnaire (GAD-7) is a seven-item measure of anxiety frequently used in clinical populations (Spitzer et al., 2006). Each item is rated on the options 0, ‘not at all’, 1, ‘several days’, 2, ‘more than half the days’, and 3, ‘nearly every day’. Total scores range from 0-21, and a higher score indicates greater symptoms of anxiety. Scores above 10 are considered to be in the clinical range. Self-report measures of the GAD-7 were used; parents completed the GAD-7 to assess their own anxiety, as did CYP aged 12 years and above.
(4) The Patient Health Questionnaire (PHQ-9) is a nine-item measure of depression commonly used in clinical practice (Kroenke et al., 2001). Each item is answered according to the frequency of symptoms (0 = not at all, 1 = some days, 2 = more than half of the days, 3 = almost every day). Total scores range from 0 to 27 and a higher score is indicative of greater depressive symptomatology and scores above 10 are considered to be in the clinical range. CYP aged 12 years and above and parents completed the PHQ-9 to assess their own depression.

Mental health outcome measures were completed at baseline and 6 months follow-up. They were collected face to face, over the phone or online by a researcher not involved in the delivery of the intervention.

At follow up, the modified Client Satisfaction Questionnaire (CSQ-8) was also used. This is a widely used measure of service satisfaction in mental health services and was adapted for use in the previous drop-in centre study (Catanzano et al., 2021; Miglietta et al., 2018)^3^. There were 7 items in total and responses were scored on a five-point scale of 1 = ‘not at all’, 2 = ‘only a little’, 3 = ‘somewhat’, 4 = ‘quite a bit’ and 5 = ‘totally’. Higher scores indicate greater satisfaction. CYP aged 12 years and older, and parents of CYP of all ages were asked to complete the CSQ-8 at follow up.

Qualitative feedback was also collected at this time via open text feedback. CYP aged 12 years and older, and parents of all CYP were asked three optional open-ended questions at the end of the follow-up survey.

(1) If the information and support you/your child received made a difference to you/your child’s mental or physical health, please give details here
(2) What was most helpful about the information and support you received?
(3) Please use the following space to share your experience of the Lucy Project and give any feedback.

### Ethics

Written informed consent was obtained for all participants aged 16 and above (who had capacity to consent). In the case of children under the age of 16 years, assent was obtained alongside parental consent. To protect participant data, all survey answers were anonymous, and data stored on a password-protected computer.

### Patient and Public Involvement

Throughout the study the Great Ormond Street Hospital Young People’s Advisory Group (YPAG) and Cambridge Parents’ Advisory Group were consulted and advised on the patient facing materials, online questionnaires and dissemination.

### Data Analysis

Data were analysed using Statistical Package for the Social Sciences, SPSS version 27.0 (IBM Corp, 2020). Analyses for between-group differences for ethnic minority and white British families were conducted for all mental health outcomes at baseline, follow up and the difference in scores before and after accessing the integrated services. Service satisfaction scores obtained at follow up, and referral pathways were also compared. T-tests or Wilcoxon signed rank tests (for non-parametric data) were used to compare the independent groups and converted into standardised effect sizes (Cohen’s d). Chi-squared analysis were conducted when comparing categorical variables.

Qualitative data were analysed using traditional inductive latent content analysis methods (Graneheim et al., 2017). The qualitative analysis process was carried out using the NVIVO software system, version 14 (Allsop et al., 2022; Lumivero, 2023).

The primary researcher, EK, first familiarised herself with the data and decontextualised findings. Responses were then divided into ‘meaning units’, individual sections based on response’s underlying meaning, as defined by the researcher (Lindgren et al., 2020). Using open coding each meaning unit was assigned a code that captured their overall concept. A coding list with illustrative examples was developed and checked against the original data to ensure consistency in coding, available in Appendix A (Bengtsson, 2016). Codes were then merged into subthemes and, through comparison with the data and quantitative findings, synthesised into overarching themes.

### Reflexivity

Reflexivity was emphasised throughout the analysis process. EK, a non-clinical MSc researcher, conducted a secondary analysis of the data, and was not involved in the initial data collection or intervention delivery process. The second senior researcher, AR, a doctoral student was directly involved in data collection and intervention delivery under the supervision of SB and RS, clinical psychologists. EK identifies as White Irish and AR as White British.

Regular meetings were held between EK and AR at the construction of subthemes and themes stage. EK’s analytic distance from the study supported her ability to focus on positive and critical feedback equally, while AR’s involvement in the study provided contextual insight and deeper knowledge of the topic. Reflexive discussions were regularly held between researchers, to critically consider how their perspectives, including their positionality as White researchers, might influence analysis. During meetings subthemes and themes were re-appraised to ensure they were reflective of the data, and researchers compared their interpretations of the data. Following agreement from both researchers, EK re-applied the final themes to the data to check fit.

## Results

### Sample

One hundred and twenty families consented to take part in the study (full sample details are presented in the main findings paper). Eighty-three families (70%) completed follow up outcomes after six months. Table 1 provides an overview of the demographic characteristics of the entire sample. For analysis, participants who did not describe their ethnicity as white British were categorised as the ethnic minority.

**Table 1.** Participant Characteristics

Participant characteristics		
*Children and young people (CYP)* (n = 120)		
Age in years (n = 120)	​	mean (sd), range
​	​	13.25 (4.31), 2-24
​	​	n (%)
Gender (n = 120)	Female	77 (64.2)
​	Male	42 (35.0)
​	Non-binary	1 (0.8)
Ethnicity (n = 120)	English, Welsh, Scottish, Northern Irish or British	79 (68.1)
​	Global majority	37 (31.9)
​	*Any other white background*	*12 (10.0)*
​	*White and Black Caribbean*	*2 (1.7)*
​	*White and Black African*	*1 (0.8)*
​	*White and Asian*	*2 (1.7)*
​	*Any other mixed or multiple ethnic background*	*6 (5.0)*
​	*Indian*	*2 (1.7)*
​	*Pakistani*	*4 (3.3)*
​	*Any other Asian background*	*1 (0.8)*
​	*Caribbean*	*1 (0.8)*
​	*Any other black, black British or Caribbean background*	*4 (3.3)*
​	*Any other ethnic group*	*1 (0.8)*
​	*Would rather not disclose*	*1 (0.8)*
​	*Missing*	*4 (3.3)*
First language (n = 120)	English	110 (94.8)
​	Other	6 (5.2)
​	Missing	4 (3.3)
*Parents* (n = 94)		
Age in years	​	mean (sd), range
​	​	42.95 (7.29), 26-58
​	​	n (%)
Gender (n = 94)	Female	87 (92.6)
​	Male	7 (7.4)
Ethnicity (n = 94)	English, Welsh, Scottish, Northern Irish or British	69 (73.4)
​	Global majority	25 (26.6)
​	*Any other white background*	*11 (11.7)*
​	*White and Black Caribbean*	*1 (1.1)*
​	*White and Black African*	*1 (1.1)*
​	*Any other mixed or multiple ethnic background*	*3 (3.2)*
​	*Indian*	*1 (1.1)*
​	*Pakistani*	*3 (3.2)*
​	*Bangladeshi*	*1 (1.1)*
​	*Any other Asian background*	*1 (1.1)*
​	*Caribbean*	*1 (1.1)*
​	*African*	*1 (1.1)*
​	*Any other black, black British or Caribbean background*	*1 (1.1)*
First language (n = 94)	English	82 (87.2)
​	Other	12 (12.8)
Number of children in house (n = 94)	​	mean (sd), range
​	​	2.30 (1.056), 1-5
Referral routes		
​	​	n (%)
​	Self-referral	54 (45)
​	Clinician referral	66 (55)

### Differences in Mental Health

There were no statistically significant differences in self or parent-reported SDQ, PHQ-9 or GAD-7 scores (at baseline, follow-up or when looking at the difference in scores before and after accessing the integrated services) when comparing White British families (n = 79) and those from ethnic minority groups (n = 37) (see Table 2). There was no difference between the ethnic groups when looking at self-reported quality of life (measured by the PedsQL).

**Table 2.** Mental Health Outcomes Comparing White British and Ethnic Minority Families

Outcomes	N	White British	Ethnic minority	Mean difference (95% CI)	p-value	D	df
Baseline measures	​	​	​	​	​	​	​
Self-reported SDQ total difficulties	90	17.88	16.42	1.46 (−0.98, 3.89)	.239	0.2	88
Parent reported SDQ total difficulties	92	16.34	17.83	−1.49 (−4.88, 1.89)	.382	−0.2	90
Self-reported PedsQL total score	103	53.04	48.78	4.26 (−2.73, 11.25)	.230	0.25	101
Parent reported PedsQL total score	94	53.37	41.15	12.22 (3.23, 21.21)	.008**	**0.63**	92
Self-reported PHQ-9	82	12.78	13.59	−0.81 (−3.66, 2.03)	.571	−0.12	80
Self-reported GAD-7	82	10.62	10.19	0.43 (−2.03, 2.89)	.727	−0.08	80
Parent-reported PHQ-9	93	7.65	9.58	−1.93 (−4.89, 1.03)	.198	−0.31	91
Parent-reported GAD-7	93	6.41	7.42	−1.01 (−3.64, 1.62)	.447	−0.18	91
Follow up measures	​	​	​	​	​	​	​
Self-reported SDQ total difficulties^a^	53	16.32	13.32	3.01 (−0.58, 6.60)	.099	0.48	51
Parent reported SDQ total difficulties^a^	48	14.56	11.67	2.90 (−3.22, 9.01)	.369	0.35	46
Self-reported PedsQL total score	57	60.37	63.29	−2.92 (−14.89, 9.06)	.628	−0.14	55
Parent reported PedsQL total score	49	57.64	73.88	−16.23 (−32.09, −.0364)	.045*	**−0.73**	47
Self-reported PHQ-9	48	9.63	11.00	−1.38 (−5.32, 2.57)	.486	−0.22	46
Self-reported GAD-7	48	7.03	9.00	−1.97 (−5.58, 1.64)	.278	−0.34	46
Parent-reported PHQ-9^a^	49	6.74	6.20	0.54 (−4.08, 5.16)	.634	0.08	47
Parent-reported GAD-7^a^	49	5.15	4.40	0.75 (−2.78, 4.28)	.308	0.15	47
Difference scores	​	​	​	​	​	​	​
Self-reported SDQ total difficulties	44	−0.74	−3.24	2.50 (−2.54, 7.53)	.323	0.31	42
Parent reported SDQ total difficulties	46	−1.14	−8.33	7.20 (−0.65, 15.05)	.071	0.69	44
Self-reported PedsQL total score	53	8.81	12.71	−3.90 (−17.41, 9.62)	.565	−0.16	51
Parent reported PedsQL total score	48	2.52	35.62	−33.11 (−54.98, −11.24)	.004**	**−1.08**	46
Self-reported PHQ-9	36	−3.19	−4.60	1.41 (−3.87, 6.69)	.591	0.18	34
Self-reported GAD-7	36	−2.86	−1.93	−0.92 (−6.34, 4.49)	.731	−0.12	34
Parent-reported PHQ-9	47	−0.24	−5.89	5.65 (−0.97, 12.28)	.093	0.64	45
Parent-reported GAD-7	47	−0.76	−6.11	5.35 (−0.01, 10.69)	.050	0.75	45

Means (M), SDs, 95% CIs around the mean difference and effect sizes (d) are shown for all data. Significant results are displayed in bold font.

SDQ: Strengths and Difficulties Questionnaire; PedsQL: Pediatric Quality of Life Inventory; PHQ-9: Patient Health Questionnaire; GAD-7: Generalized Anxiety Disorder.

^*^p < .05; **p < .01.

^a^Mann Whitney test used for non-parametric data.

There were however differences when comparing the parent-reported PedsQL at all time points. At baseline, parents from ethnic minority backgrounds reported significantly lower quality of life scores for their child, 41.15 (SD = 19.02) compared to White British parents, 53.37 (19.52), t (92) = 2.7, p < .01, d = 0.63. At follow up, this changed, and parents from ethnic minority families reported a significantly higher quality of life for their child, 73.88 (15.43), compared with White British parents, 57.64 (23.58), t (47) = −2.06, p < .05, d = −0.73. The exploratory analysis also indicated a difference in mental health change scores, with ethnic minority families showing greater improvements after accessing the service, *t* (46) = −3.05, *p* < .01, *d* = −1.08.

### Satisfaction With Service (CSQ-8)

Seventy families completed the CSQ-8 at follow up (58.33%). Across both parent-reported and CYP-reported scores there were no significant differences between ethnic groups across total satisfaction scores (Self-reported: Z = −1.27, p > .05; Parent-reported: Z = -.594, p > .05) (see Table 3 and results per individual question are available in Appendix B - Figure 1 and 2.).

**Table 3.** Satisfaction Scores Comparing White British and Ethnic Minority Families

Satisfaction	N	White British	Ethnic minority	Mean difference (95% CI)	p-value	D	df
Self-reported CSQ-8 total score	41	26.07	22.15	3.92	.134	.51	39
Parent reported CSQ-8 total score^a^	29	27.22	27.50	−0.28	.550	.03	27

CSQ-8: Modified Client Satisfaction Questionnaire.

^a^Mann Whitney test used for non-parametric data.

### Differences in Referral Routes

Chi-squared analysis was conducted to compare referral routes across ethnic groups (n = 116) (Table 4). There was a significant difference between referral routes, with significantly more CYP from White British families accessing the drop-in services via self-referral, X (1) = 8.32, p = .004.

**Table 4.** Referral Route Comparisons Between White British and Ethnic Minority Families

Referral routes		
​	White British CYP	Global majority CYP
Self-referral	44	10
Clinician referral	35	27
​	​	X^2^ = 8.32 p = .004**

X^2^ – Chi squared test; p = significance level.

**p < .01.

### Qualitative Feedback

104 participants provided written open-text feedback, which generated 242 lines of text for analysis. Overall, participants across all ethnic backgrounds had a positive experience with the service, describing The Lucy Project as *“lifechanging”,* feeling that it “*overmet [their] needs”* and was *“outstanding”*.

Data were analysed using inductive latent content analysis (reference) which generated 96 codes, made up of 11 categories and eventually five themes were derived. These were: (1) “Life changing” support; (2) Having someone to talk to; (3) Becoming ones’ own therapist; (4) Parental service experiences and (5) General reflections. A breakdown of each theme is available in Table 5.

**Table 5.** Themes

Theme	Frequency of references	Description of theme	Sub-theme	Illustrative quotes
Life-changing support	89 references - 21.71% thematic coverage of text	The drastic improvements to psychosocial wellbeing experienced by participants. Characterised by increased independence, autonomy and general engagement in everyday life.	Improved CYP well-being	*“I pretty much never worry about lumps or aches any more, and I feel more confident and happy generally. I also get angry less easily and frequently.”*
				- Non-binary young person, 16, white-British
			Improved everyday functioning	*“ It helped me face my fears on talking to people and going crowded places”*
				- Female young person, 18, any other black, black British or Caribbean background
			Improved mind-set	*“She has completely changed (young person)’s mindset in regards to her worries and anxieties […] She is now more confident to approach situations with a positive mental attitude of “I want to do this for myself”*
				- Mother, 37, white-British
Having someone to talk to	84 references - 19.22% thematic coverage of text	The emotional and supportive factors that facilitated therapeutic outcomes – with an emphasis on the importance of positive therapist relationships.	Receiving emotional support & positive therapist attributes	*“[Therapist] had a warm and welcoming demeanour, which made us feel comfortable. [Therapist] listened attentively to our concerns and provided us with helpful feedback”*
				- Mother, 38, any other mixed or multiple ethnic background
			Positive therapist-relationship	*“[Therapist] was really nice and understood me […] She is amazing and will be missed”*
				- Female young person, 14, white-British
Becoming ones’ own therapist	58 references - 15.39% thematic coverage of text	The experiences of participants who received the LI-CBT intervention. Feedback focused on positive experiences with CBT techniques and approaches. Specifically, participants reported appreciating factors such as psychoeducation and practical techniques they can use to manage worries and the general challenges of everyday life, normalising their experiences.	Becoming ones’ own therapist	*“ The most helpful for me was the techniques with my panic attack and my anxiety”*
				- Female young person, 12, any other black, black British or Caribbean background
			The guided self-help process	*“The booklets and explaining - puts it into perspectives, makes me realise I’m normal, I’m not weird or anything- put my mind at ease. Explained situations I can relate to - helpful”*
				- Male young person, 17, any other black, black British or Caribbean background
Parental service experiences	37 references - 13.23% thematic coverage of text	Parents’ experiences with the service. Characterised by improvements in parental MH after observing improvements in child well-being, experiences engaging with parent-led interventions and parents’ perception of parental supports available within the service.	Improved parent mental health	*“The support [CYP] received helped her mental health and in turn my own mental health.”*
				- Mother, 48, white-British
			Parenting supports	*“It was reassuring to have regular discussions about my son’s behaviour and techniques to improve it. It was useful to be able to offload about difficulties we were having and having a sounding board on ideas on how to manage his behaviour.”*
				- Mother, 38, white-British
General reflections	138 references - 31.94% thematic coverage of text	Participants’ reflections on their experiences and satisfaction with the service, and how accessible and useful it was. Participants reflected on the relief of accessing support, particularly after trying and failing to access services previously. Further, explores areas participants enjoyed about the service and areas they feel could be improved upon.	An accessible service	*“ We felt that we’ve been listened [to] and willingness to refer (CYP) to the right service, after so many years of battling and being ignored and not listened by some professionals and services.”*
				- Mother, 43, any other white background
			General satisfaction	*“It’s been an amazing experience I’ve learned new things over the past few months and I’m becoming a better me.”*
				- Female young person, 12, any other black, black British or Carribean background
​	​	​	Recommendations for improvement	*“Perhaps depending on circumstances - could the criteria be lowered… these are children who have lots of appointment going to the hospital is draining, could everyone get some support? Could only [be] one call”*
				- Mother, 30, any other white background

Although a formal qualitative comparison was not done between ethnic groups, it was noted that some themes came up more for families from ethnic minority backgrounds (e.g. of all 28 quotes from ethnic minority CYP, 13 referenced low-intensity CBT processes). Illustrative quotes are displayed below.

## Discussion

The main aim of this study was to understand whether drop-in services are an accessible and acceptable way of providing psychological support for CYP with LTCs and their families. To achieve this, the research examined ethnic differences in service usage and outcomes, alongside qualitative feedback on families’ experiences of the service. Results indicated that drop-in services at paediatric healthcare settings are both accessible and acceptable.

To explore accessibility, mental health symptoms, access routes and service satisfaction were compared across the different ethnic groups. Results indicated that families from ethnic minority backgrounds did not significantly differ from White British families in measures of emotional and behavioural symptoms, anxiety or depression at baseline, 6-month follow up, or when comparing differences before and after accessing the drop-in services. Additionally, there was no significant difference in satisfaction scores when comparing these groups. Overall, significant improvements in emotional and behavioural symptoms, reductions in anxiety and depression, alongside high degrees of satisfaction with the service, were observed across ethnic backgrounds.

The only significant mental health difference identified between the ethnic groups was the parent-reported quality of life at all time points. At baseline, parents from ethnic minority backgrounds reported significantly lower quality of life scores for their child when compared to White British parents. At follow up, this changed, and parents from ethnic minority families reported a significantly higher quality of life for their child. There may be some reasons for this, firstly the baseline measure may reflect that initially these CYP may have started with a higher burden of unmet needs, delayed diagnosis, or untreated symptoms. Regarding the positive improvements over 6 months, this could reflect the benefit of the intervention itself (even if starting from a lower baseline), and/or the benefit of being able to access any support, where previously this was difficult. Furthermore, as no differences were found when comparing self-reported quality of life, it is difficult to determine whether lower parent-reported PedsQL scores are a true reflection of CYP well-being. Research has previously shown that discrepancies between parent and CYP reported measures are pronounced in ethnic minority groups (Roberts et al., 2005).

When referral routes were compared between White British families and those from global majorities, there were significantly more White British families who came via self-referral, in line with previous IAPT research (Harwood et al., 2023). This may reflect differences in awareness of available services, with White British families perhaps more familiar with different options, or could be attributed to different cultural perceptions of help-seeking (Bhavsar et al., 2021; Memon et al., 2016). Asking for help may be perceived as a sign of failure or weakness, and thus directly self-referring for support may not feel comfortable (Jacobs & Pentaris, 2021).

When compared with nationwide CAMHS data, families from non- White British, ethnic minority backgrounds were over-represented in recruitment. It is reported elsewhere that 21% of CAMHS users identify as being from non White-British backgrounds (NIHR PenARC, 2022), whereas this study had a higher proportion of CYP from ethnic minority groups (32%). This is in line with findings from the original drop-in service study at one hospital, which included 39% of their sample from ethnic minority families (Catanzano et al., 2021), and may suggest that the drop-in model as an effective way to increase access to mental health support amongst ethnic minority populations. As explored above, this may not be explained solely via the self-referral option (as this study found significantly more White British families joining the study via this route). This could instead highlight other advantages of this integrated model such as awareness and support from physical health teams to share information and refer families directly and the flexibility around intervention modality to suit family’s needs (e.g. sessions could take place online to reduce in-person visits). The higher proportion of ethnic minority CYP using the service may also reflect the higher likelihood of CYP in this group to develop LTCs compared to those from White-British backgrounds (Ekezie et al., 2023; Hayanga et al., 2024).

### Acceptability

To explore acceptability, qualitative feedback from open-text questions were analysed and satisfaction scores were compared. There were no differences between the ethnic minority and White British groups when comparing service satisfaction from both CYP and parent participants. This is particularly promising as previous research has associated ethnicity with lower levels of physical and mental healthcare satisfaction compared to their white counterparts (Cai & Robst, 2016; Pinder et al., 2016). When analysing the open-text feedback, families from all cultural backgrounds reported feeling grateful for the service, and described it as life-changing, helpful and supportive.

CYP from ethnic minority backgrounds highlighted the benefits of low intensity CBT related processes, such as breathing techniques and psychoeducation, which normalised experiences and promoted self-regulation. Such approaches may have helped ethnic minority CYP reduce self-stigma surrounding mental health. CYP across ethnicities also developed strong therapeutic relationships, and no references were made to cultural issues or lack of sensitivity from CBT practitioners, indicating a culturally competent service. Our findings demonstrate that therapeutic relationships are feasible to foster within a low intensity treatment model and aligns with previous findings that valued therapeutic relationships are important for positive mental health outcomes for CYP with LTCs (Ludlow et al., 2020; Moore et al., 2019; Shaw et al., 2019). Positive experiences with the low-intensity CBT process and improved mental health outcomes reported across ethnicities indicate low-intensity CBT is an acceptable and effective form of mental health care for CYP with LTCs, aligning with previous research (Catanzano et al., 2021a).

Findings revealed that drop-in services were perceived as an accessible form of mental health support for families, many of whom described having previously sought support elsewhere unsuccessfully. Timely access to care is crucial for CYP with LTCs, as long-wait times are associated with increased negative psychological outcomes such as self-harm, and risk of negative thought processes that one is not “deserving” of care (Jones et al., 2022; Punton et al., 2022). In this study CYP described mental health improvements primarily in terms of both their everyday functioning and general mindset.

### Strengths and Limitations

This study has several strengths. It utilised a mixed-methods approach, capturing service outcomes and process factors, which are imperative to comprehensive service evaluation (Lindsay, 2013). The paper aimed to understand the accessibility and acceptability of these new services, both are key aspects of implementation (Peterson et al., 2023). Accessibility was best explored using the quantitative data (e.g. referral routes) and acceptability could be explored through analysing the open-text feedback. Mixed methods research offers a holistic insight into families’ experiences and can answer both “how” and “why” questions. This methodology was used to provide more depth and breadth, beyond what quantitative or qualitative data alone could provide, and is recommended for health research (Wasti et al., 2022).

The study included participants from different healthcare settings across England and had a high recruitment rate. Measures of mental health and satisfaction used standardised, validated questionnaires and where statistical differences were found between ethnic groups, the effect sizes were medium to large. This said, these findings should be interpreted with caution given the exploratory nature of the analysis and limited statistical power.

Further, all non-White British participants were grouped together for analysis, as the numbers in each ethnicity group was too small for meaningful comparisons. This may have concealed nuances in how different demographic groups both accessed psychological care, and the effect it had on mental health outcomes. In addition, the analysis did not correct for multiple comparisons and a formal power analysis was not completed as this was an exploratory study. It should also be acknowledged that participants may have been more motivated to complete follow up measures and provide feedback if they had positive experiences or received their favoured intervention through the project, creating potentially biased responses.

In addition, the majority of parental respondents were Mothers (92.6%), limiting the extent to which our findings can be applied to Fathers. While this is reflective of the wider research area, inclusion of Fathers’ experiences is important, due to the impact of having a child with a chronic condition on the entire family unit (Spurr et al., 2023).

Finally, as noted in the introduction, the term “global majority” is now greatly preferred as an inclusive, de-colonial alternative to “ethnic minority” (Lee et al., 2023). For this study, the way in which the comparisons were conducted meant that global majority was not an accurate or appropriate term to use, but future work should seek to use language that is more inclusive and preferred.

## Conclusion

Overall, the study suggests that integrated mental health services delivering low intensity interventions are both accessible and acceptable to CYP with LTCs. Findings indicate that this model may be particularly effective in reaching families from ethnic minority groups within paediatric healthcare settings. There were similar mental health outcomes and high satisfaction among all demographic groups which supports the NHS strategy of integrating mental and physical healthcare. It is hoped that these findings can be shared widely so other settings may roll-out similar services to ensure CYP with LTCs receive the support they need regardless of ethnicity.

## Figures and Tables

**Figure 1.**
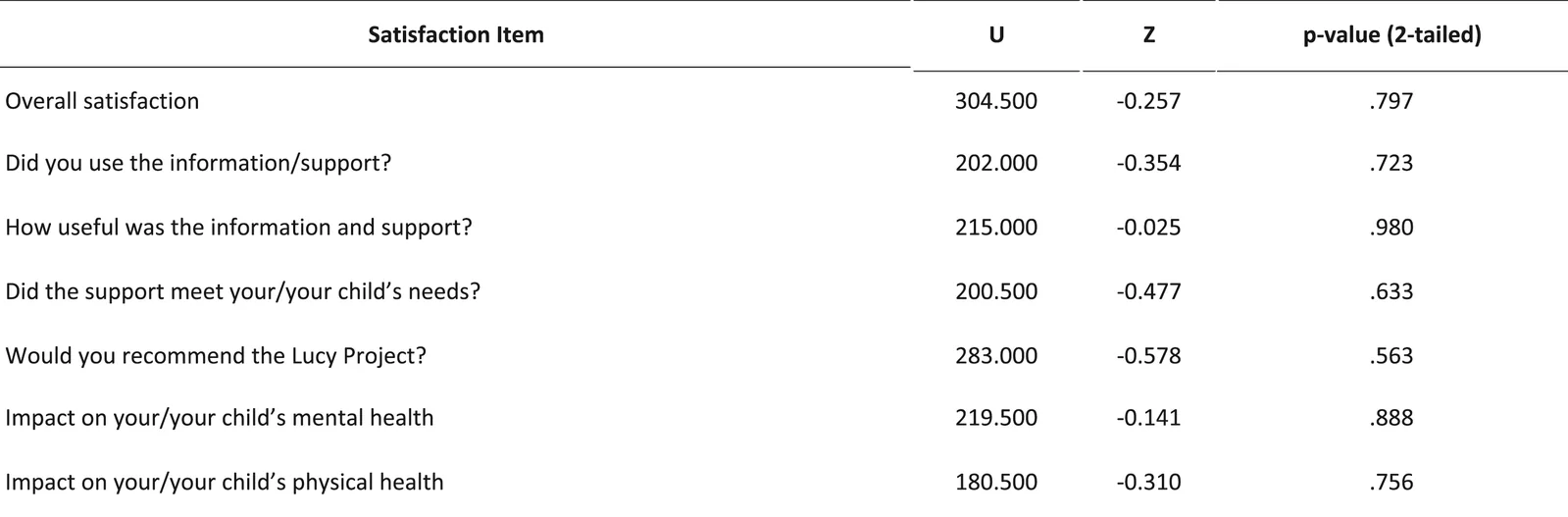
Mann–Whitney U test results comparing satisfaction between ethnic minority and non-minority parent participants

**Figure 2.**
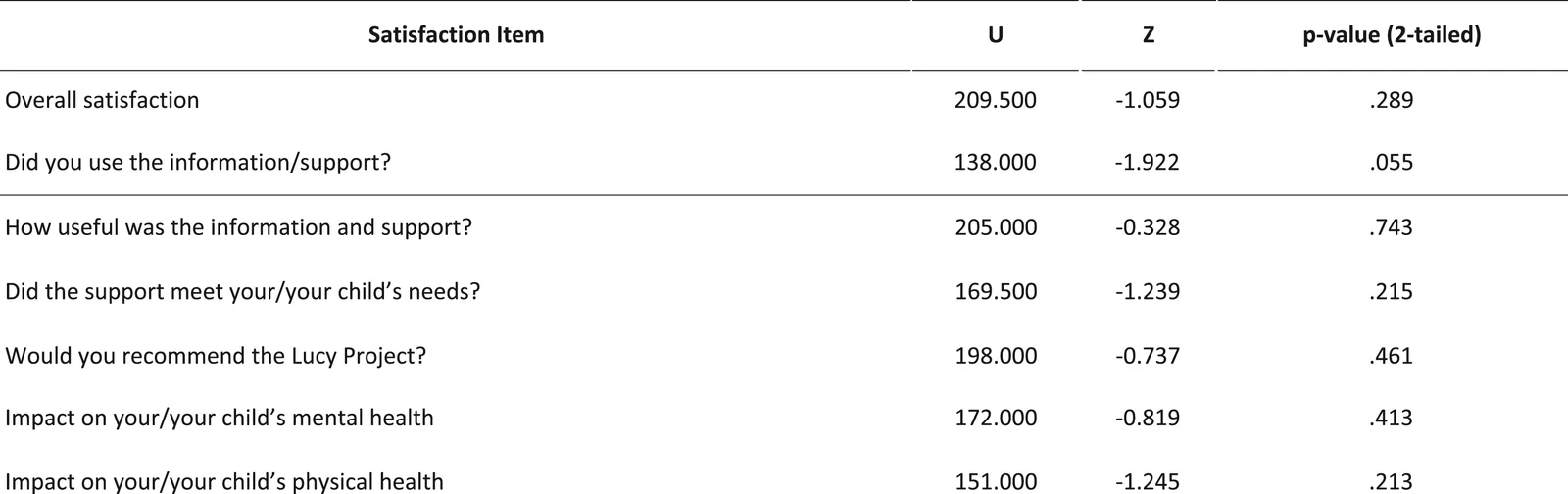
Mann–Whitney U test results comparing satisfaction between ethnic minority and non-minority CYP participants

## Notes

1. It is acknowledged that the term “global majority” is now preferred over “ethnic minority” (Lee et al., 2023), however for this analysis, families who did not identify as White British, could not always be accurately described as from the global majority. This is discussed in our study limitations.

2. 1 - White British; 2 – Irish; 3 - Any other White background; 4 - White and black Caribbean; 5 - White and black African; 6- White and Asian; 7 - Any other mixed background; 8 – Indian; 9- Pakistani; 10 – Bangladeshi; 11 - Any other Asian background; 12 – Caribbean; 13 – African; 14 - Any other Black background; 15 – Chinese; 16 - Any other ethnic group; 17 - Prefer not to say.

3. Questions regarding the kind of service, amount of help, and whether participants would return were removed, and questions on improvement to mental and physical health were added.

## Appendix

### Appendix A*:* Final Coding List

**Table:**

Theme	Code	Number of references	Illustrative quote
A life-changing service	Reduced health anxiety	4	*“(CWP) made me feel less anxious about my epilepsy.”*
​	Improved child MH	47	*“I believe my child has made significant improvements to his mental health in the last 6 months.”*
​	Feeling happier	7	*“I feel less sad.”*
​	Reduced anxiety	21	*“I feel so much less anxious all the time.”*
​	Improved confidence	11	*“I was able to grow in my confidence.”*
​	Boost to child	2	*“As a parent I felt that the project gave [child] a huge boost.”*
​	Improved sense of self	3	*“I’m becoming a better me.”*
A life-changing service	Improved everyday functioning	13	*“Transformed his life, doing things he never would have dreamt off… He’s been going into shops, got a lift back with people, going for a walk by himself on holiday… He’s got a new sense of freedom… He would never have done that…”*
​	Ability to overcome challenges	7	*“I was able to go to prom despite my anxieties.”*
​	Improved school activities	9	*“[child] navigated 6 weeks of GCSE exams with practically no stress or anxiety, which was a fantastic result, given how she had been during previous exams.”*
​	Improved social interactions	7	*“It made me less socially anxious around other people.”*
A life-changing service	Increased independence	2	*“The support received has helped us manage [child]’s separation anxiety and she is now in much more positive mind frame about being left at bedtime and also being able to go out and leave her.’*
​	Improved mindset	12	*“She has completely changed (child)’s mindset in regards to her worries and anxieties.”*
​	Eased mind	10	*“[The support] put my mind at ease.”*
​	Given perspective	2	*“The booklets and explaining - puts it into perspectives.”*
​	Increased openness	4	*“She was a bit more open and tried new things.”*
​	Improved physical health	4	*“Her physical symptoms have also subsided a lot.”*
A life-changing service	Improved sleep	4	*“There was further support on my daughter’s problem with insomnia. The nurse and doctor spent time with her and me and discussed options. They have prescribed melatonin which helps her sleep well.”*
​	Visible improvement	2	*“I could see how much he benefited from it.”*
​	Children unaware of impact	2	*“I believe [child] is not aware of how much this has helped him.”*
​	Hope for maintained improvements	1	*“I hope she will continue to so this now that the sessions have ended.”*
​	Lack of change	2	*“It would have been good however after so many sessions to have witnessed an improvement in my daughters ability to deal with her acute IBS episodes.”*
A life-changing service	Lack of longevity	2	*“[child’s ]mental health did improve with the sessions although managing to regulate it and stay on top of it has become a lot for [child] at present and she’s mentally not in a good place.”*
​	Remaining difficulties	4	*“We tried different techniques to support [child]’s anxiety surrounding his needle but unfortunately [child]anxiety is still very much present it’s an on going battle.”*
Having someone to talk to	Acceptance	1	*“Its helped [child] accept her pain/migraine attacks.”*
​	Re-assurance	5	*“It was so lovely having someone to talk to and be reassured.”*
Having someone to talk to	Feeling listened to	8	*“The lady that I spoke to was very kind she acknowledged and heard my voice.”*
​	Normalising	9	*“Puts it into perspectives, makes me realise I’m normal.”*
​	Patience	1	*“I love how everyone so far has been very patient with me because I’m quite a handful.”*
​	Processing trauma	2	*“It’s good to know there is support for young people that have live through a harrowing experience and struggle to process the aftermath.”*
​	Rationalising	1	*“[Child] being able to speak with someone that can rationalise.”*
​	External support	9	*“We saw a difference in [child] just to have adult support and backing outside of the family home - at school for example.”*
Having someone to talk to	Having someone to talk to	8	*“Having someone to talk to was nice.”*
​	Regular available support	5	*“It was nice to have someone to talk to weekly about how I felt.”*
​	Support when needed most	3	*“It came at a very difficult time and helped steady him when he needed it most.”*
​	Team of support	5	*“The team have been very supporting, kind and helpful.”*
​	Validation	5	*“She validated some of the feelings I had/was having, which was extremely helpful.”*
​	Liking therapist	6	*“I love working with [therapist] I miss her.”*
​	Positive therapeutic experience	2	*“My daughter appeared to enjoy the sessions.”*
​	Therapist attributes	16	*“[Therapist] was brilliant and friendly… Above and beyond…”*
Having someone to talk to	Personalised	1	*It was good how it was personalised to my child’s needs.*
​	Will miss support	4	*“She is amazing and will be missed.”*
​	Safe environment	4	*“I loved how I was constantly reminded that I am in a safe space*.*”*
​	Feeling understood	1	*“[Therapist] was really nice and understood me*.*”*
​	Be open about difficulties	6	*“It helped me to start opening up about how I feel*.*”*
​	Therapist empathy	1	*“Being able to […] feel how life is impacting a severely disabled child and their family*.*”*
​	Difficulty opening up	1	*“My son finds it difficult to speak about his issues with a psychologist*.*”*
The LI-CBT process	Ability to cope	4	*“Special thank you for (therapist) as she really helped me to learn helpful coping mechanisms and to manage situations better*.*”*
The LI-CBT process	Advice not useful	3	*“The support and advice given I don’t feel as though it help much*.*”*
​	Resources explained well	2	*“To have (cwp) explain sections of the book to me [was most helpful]*.*”*
​	Improved understanding of emotions	8	*“The Lucy Project helped me gain a better understanding about what I was feeling, why I was feeling it and how I could resolve it.”*
​	Psychoeducation on brain-body connection	2	*“I understand more about my brain and body and how to notice little throngs that can affect my mood.”*
​	Make CBT more approachable	1	*“CBT - at the start explain it more (more examples of people it has helped, showing like some example ladders)*.”
The LI-CBT process	Initial ambivalence to CBT	1	*“At first i was thinking how is this gonna help me - it really surprised me - it really did! the later i got, the more it helped! at the start i was less motivated*.”
​	Not suitable for CBT	4	*“For my specific case the cbt didn’t work at all*.”
​	Relevant resources	1	*“The information from (therapist) was age appropriate, helpful and well explained.”*
​	Unable to use advice	2	*“Some good advice was given however, I feel like I am the one holding myself back by not taking on all the good advice*.”
​	Use of techniques	23	*“I was able to grow in my confidence and have a mind settled by using the techniques I was taught.”*
​	Breathing techniques	7	*“I loved how I was introduced to new techniques and breathing techniques that I feel like are not widely known by other teens*.”
The LI-CBT process	CBT-specific resources	2	*“The homework and practical tasks given allowed me to practise and regain confidence*.”
​	Use of booklets	4	*“The booklets and explaining - puts it into perspectives, makes me realise im normal, im not weird or anything- put my mind at ease*.*”*
​	Self-regulation	15	*“Helped me control my worrying on a level that I would be able to use in everyday life*.*”*
​	Useful advice	5	*“[child] has found the advice useful and supportive*.*”*
​	Ways to manage	7	*“It helped me manage stressful moments (e.g. with breathing techniques) which made things generally easier to deal with*.*”*
Parental service experiences	Desire for more parental support	3	*“It would be useful if parents could have the same support from UCLH. I liked that I was able to talk about my experience of [child ]time in hospital and her illness, but I wasn’t able to have regular chats as [child] was able to*.*”*
​	Family involvement	1	*“It was great as a family to have the time to sit together and talk through issues*.*”*
​	Information openly shared with parents	2	*“All support and information was readily shared at every step. Even upon the end of sessions the information and guidance on what to do if further support was needed was very comprehensive and helpful.”*
​	Poor access to information	1	*“As a parent, I did not receive any information or resources.”*
Parental service experiences	Desired framework to continue progress	1	*“It would have been useful to have received at least a summary outlining the key areas of improvement and what I can do moving forward to continue with the psychologists good work*.*”*
​	Improved family support for CYP	1	*“It has really helped us as a family, learn how to help (child) too*.*”*
​	Lack of parental support elsewhere	1	*“I guess it just brought home how bad (and how “quick fix”) the mental health provision is for parents*.*”*
​	Improved parent MH	8	*“The support [child] received helped her mental health and in turn my own mental health, as I knew she was benefitting from the support she received*.*”*
Parental service experiences	Parent relief	4	*“[child] felt supported and felt he could cope better. That took the pressure of me, worrying he might do something to himself*.*”*
​	Parenting supports	10	*“It was reassuring to have regular discussions about my sons behaviour and techniques to improve it. It was useful to be able to offload about difficulties we were having and having a sounding board on ideas on how to manage his behaviour*.*”*
​	Support taking the right steps	4	*“She’s been able to talk us through the steps to support [child]*.”
​	Techniques/supports parents are already aware of	4	*“The techniques I learnt were not new*.*”*
General reflections	Accessibility	6	*“Valuable access to support that we may not otherwise have received so quickly*.
​	Wait-list issues elsewhere	4	*“Even now, camhs have us on a 18 month waiting list… To thinkof not having an appt til he’s 8*.*”*
​	Accommodating	2	*“The nurse was excellent - very affirming of my daughter, went out of her way to accommodate the school day*.*”*
​	Preference for alternative support	2	*“However, the thing that has most helped her has been private acupuncture. She’s found it wonderfully relaxing*.*”*
​	Ambivalent response to the service	2	*“It’s OK I guess*.*”*
​	Assessment service experiences	4	*“The psychological assessment that my daughter received was helpful*.*”*
General reflections	Referral service experiences	13	*“Referrals put in place so that was helpful*.*”*
​	Clash with CAMHs	3	*“Its an interesting project however we did not get a chance to use it much since we were working with CAMHS*.*”*
​	General satisfaction	35	*“We are extremely happy and satisfied with the project and especially with our Psychologist.”*
​	Feeling grateful	17	*“Thank you so much for all the help and support with [child]. It really is hugely appreciated and we’re extremely grateful.”*
​	A helpful service	51	*“I thank the Lucy project and believe it has really helped me.”*
​	Would recommend to others	4	*“I’d definitely recommend to others in need.”*
General reflections	Support in the battle to be helped	3	*“I feel it has been another tool in the arsenal of the lengths I’m trying to get through to get an assessment for my son and seek answers.”*
​	Support while waiting for further help	2	“*Knowing [child] had someone to contact at the Lucy Project until CAMHS came through.”*
​	Hopeful for future support	2	*“Would love to see the benefits of engaging with this project to help (child)*.*”*
​	Keeping in touch	2	*“[Therapist] was great with keeping in touch and touching base with my child [child]*.*”*
​	Were not seen by service	6	*“We filled out the qn. Attended the assessment and then were told it had stopped. No help received.”*
​	Only in initial stages of support	1	“*We have just started support so I don’t know the outcome at the moment.”*
General reflections	Service could not meet CYP’s needs	8	*“[child] needs couldn’t be met through project. Was deemed not appropriate.”*
​	Long wait-time for support	4	*“It took quite a while for us to be contacted by the lucy project after our first session(and in that time we were contacted by CAMHS and started working with them)*.*”*
​	Less stringent criteria	1	*“Perhaps depending on circumstances - could the criteria be lowered… These are children who have lots of appointment going to the hospital is draining, could everyone get some support? Could only [be] one call.”*
​	Preference for in-person support	1	*“Altho it would have been better to be face to face…”*
​	Desire for further support	5	*“We’re very grateful for this opportunity and wish it could carry on!”*
​	Desire for more flexibility	2	*“Support outside of work hours would be great.”*
General reflections	Desire for wider range of support	3	*“I feel that it would be more helpful if there was a wider range of things to speak about.”*
